# Incremental value of three-dimensional echocardiography for evaluating left atrial function in patients with coronary slow flow phenomenon: a case control study

**DOI:** 10.1186/s12947-020-00189-z

**Published:** 2020-02-13

**Authors:** Jun Li, Yonghuai Wang, Cuiting Zhao, Qing Zhu, Guangyuan Li, Jun Yang, Dalin Jia, Chunyan Ma

**Affiliations:** 1 Department of Cardiovascular Ultrasound, The First Hospital of China Medical University, No. 155 Nanjingbei Street, Shenyang, Liaoning 110001 People’s Republic of China; 2 Department of Cardiology, The First Hospital of China Medical University, Shenyang, Liaoning People’s Republic of China

**Keywords:** Coronary slow flow phenomenon, Left atrial, Three-dimensional, Echocardiography

## Abstract

**Background:**

Coronary slow flow phenomenon (CSFP) involves the delayed opacification of the coronary distal vessel, in the absence of an obstructive lesion in the epicardial coronary artery during angiography. Since the link between left atrial (LA) function and decreased left ventricular function is still unclear, we evaluated LA function using real-time three-dimensional echocardiography (RT3DE) in patients with CSFP, and subsequently determined the incremental value of RT3DE.

**Methods:**

This study enrolled 60 patients with CSFP and 45 control subjects. CSFP was diagnosed based on thrombolysis in myocardial infarction frame count (TFC). The LA phasic volume and function was evaluated by both two-dimensional echocardiography (2DE) and RT3DE.

**Results:**

The LA maximal volume (Vol_max_), pre-systolic volume (Vol_p_), and minimal volume (Vol_min_) increased, but LA total and active ejection fraction decreased in patients with CSFP. Based on our results, Vol_max_, Vol_p_, Vol_min_, and LA total and active ejection fraction correlated with TFC, and with the number of arteries involved. The LA total ejection fraction by RT3DE was the only independent predictor for CSFP (odds ratio, 0.64 [95% confidence interval, 0.49–0.83]; *P* = 0.001). Also, the LA total ejection fraction by RT3DE demonstrated good predictive power for CSFP, with a cut-off value of 54.15% (area under curve, 0.85; sensitivity, 84%; specificity, 83%).

**Conclusions:**

The LA reservoir and contractile function decreased in the patients with CSFP and correlated with coronary flow rate and with the number of arteries involved. The LA total ejection fraction by RT3DE can independently predict CSFP, and RT3DE demonstrated incremental value for evaluating LA phasic function in the patients with CSFP compared to 2DE.

## Background

Coronary slow flow phenomenon (CSFP) is characterized by the delayed opacification of the coronary distal vessel in the presence of an apparently “normal” epicardial coronary artery during angiography [1, 2]. It has been reported that CSFP may lead to acute coronary syndrome and increase the risk of acute myocardial infarction or malignant arrhythmia [3, 4]. Moreover, several previous studies have demonstrated that left ventricular (LV) systolic and diastolic functions decreased, although there was no evidence of stenosis of the coronary artery in patients with CSFP [5, 6]. Therefore, patients with CSFP should be placed under close clinical monitoring.

Left atrial (LA) function includes reservoir, conduit, and contractile function, and plays a close interaction with LV function [7]. Specifically, LA reservoir function depends on LA compliance and LV systolic function, while LA conduit function is based on LV diastolic function and LA contractile function relies on LA contractility and LV end-diastolic pressure. However, the means by which changes in LA function are associated with decreased LV systolic and diastolic function in patients with CSFP is not well understood. It has been revealed that LA function is a prognostic marker for diverse cardiovascular conditions and closely correlates with disease severity [8, 9]. Therefore, the evaluation of LA function in patients with CSFP may provide valuable information for the determination of prognosis, severity, or even may assist with the assessment of therapeutic efficacy.

Echocardiography is the most common non-invasive imaging technique used to quantify LA volume and function, and the biplane method of two-dimensional echocardiography (2DE) is frequently used. However, the measured value of 2DE may be affected by the significant geometric assumptions that exist about LA nonsymmetrical shape or foreshortening of LA cavity in the apical views. Real-time three-dimensional echocardiography (RT3DE) is a useful tool for assessing LA volume and function, with good feasibility and reproducibility, and it has been reported that the LA measurements by RT3DE are more strongly associated with cardiac magnetic resonance imaging than those by 2DE [10, 11].

In view of the foregoing discussion, we conducted this study to evaluate LA function by RT3DE in patients with CSFP, and to determine the incremental value of RT3DE compared to 2DE.

## Methods

### Study population

One hundred and sixty patients with normal or near normal epicardial coronary arteries (no coronary stenosis exceeded 40%) were selected from a population of patients who were submitted for elective coronary angiography, due to suspected coronary heart disease from the Cardiology Department of our hospital from January 2016 to December 2018.

The patients with thrombolysis in myocardial infarction (TIMI) frame count (TFC) exceeding 27 frames in at least 1 coronary artery was assigned to the CSFP group. The exclusion criteria included the following: coronary aneurysm-like dilation or dissection, positive exercise test, history of myocardial infarction, previous coronary intervention for coronary heart disease, myocardial bridge, valvular heart disease, congenital heart disease, primary cardiomyopathy, LV ejection fraction (LVEF) < 52% in males or < 54% in females, uncontrolled primary or secondary hypertension, arrhythmia, history of ischemic or hemorrhagic cerebrovascular disease, chronic obstructive pulmonary disease, severe malnutrition, liver or kidney dysfunction, tumor, acute or chronic infection, autoimmune disease, thyroid dysfunction, and poor quality of coronary angiography or echocardiography. The remaining patients with TFC not exceeding 27 frames in all coronary arteries were assigned to the control group with the same exclusion criteria. In the end, our study sample included 60 patients with CSFP (36 males and 24 females; 56.5 ± 8.8 years old) and 45 control subjects (21 males and 24 females; 55.3 ± 8.4 years old) after excluding 55 patients.

Written informed consent was obtained from all patients before enrollment. The study protocol was approved by the China Medical University Ethics Committee and was conducted in accordance with the ethical guidelines of the 1975 Declaration of Helsinki.

### Coronary angiography and TFC

Coronary angiography was performed using the standard Judkins method. The same contrast medium was used in all patients. The image acquisition speed was 30 frames. Coronary blood flow velocity was assessed by TFC. TFC is the number of TIMI frames from the moment that the contrast agent occupies the width of the proximal coronary artery to the moment in that the contrast agent reaches the distal mark of each coronary artery [1]. The frames of left anterior descending coronary artery (LAD) and left circumflex coronary artery (LCX) were counted in the right anterior oblique and foot position. The frames of right coronary artery (RCA) were counted in the left anterior oblique and head position. Since LAD is longer than LCX and RCA, TFC is divided by 1.70 to obtain a corrected frame count (cTFC). The mean TFC for each patient is the average value of TFC among the 3 major coronary arteries. Evaluation of coronary angiography data for all subjects was performed by 2 independent experienced cardiologists, and a third observer resolved any disagreements.

### Echocardiography

Echocardiographic examination was performed within 72 h after coronary angiography using a iE33 ultrasound system (Philips Medical System, Andover, MA, USA) with an S5–1 transducer and X3–1 matrix-array transducer (1–3 MHz), in accordance with the recommendations of the American Society of Echocardiography (ASE) [12]. Two-dimensional echocardiography images, including at least 3 consecutive cardiac cycles, were stored in a cineloop format for offline analysis. Real-time three-dimensional echocardiography images were obtained and stored in full volume data sets using an apical approach, avoiding the use of frame rates lower than one third of heart rate based on standard recommendations [13].

According to the recommendations for cardiac chamber quantification from ASE, LVEF, and LV, global longitudinal strain (GLS) was measured to assess LV systolic function [12]. Meanwhile, according to the recommendations for evaluation of left ventricular diastolic function from ASE, mitral early diastolic flow velocity (E), late diastolic flow velocity (A), early diastolic annular velocity (e’), and tricuspid regurgitation velocity were measured, and then mitral E/A and E/e’ were calculated to assess LV diastolic function and LV filling pressures (LVFP) [14].

### LA volume and function by conventional echocardiography

The following measurements were performed using the biplane Simpson method: LA maximal volume (Vol_max_) at the onset of mitral opening, pre-systolic volume (Vol_p_) just before the P wave on surface electrocardiogram, and minimal volume (Vol_min_) at the onset of mitral closure. The LA total emptying fraction was calculated as (Vol_max_ – Vol_min_)/ Vol_max_ × 100. The LA active emptying fraction was calculated as (Vol_p_ – Vol_min_)/ Vol_p_ × 100. The LA passive emptying fraction was calculated as (Vol_max_ – Vol_p_)/ Vol_max_ × 100.

### LA volume and function by real-time three-dimension echocardiography

The RT3DE data was analyzed using QLAB software system (Philips Medical Systems, Andover, Massachusetts). The LA models were created by marking 5-points on the atrial surface of the mitral annulus (anterior, inferior, lateral, and septal), with the fifth point at the apex of the LA. Once the points were completed, the endocardial border was automatically delineated, and LA volume was obtained throughout the heart cycle. Finally, a 3D LA model and LA volume-time curves were generated. Subsequently, the LA phasic volume and function were computed. In some patients, manual modifications were necessary to correct the automatic tracings, and points assumed to be within the pulmonary vein ostia, or LA appendage, were excluded from the measurement.

### Intra- and Interobserver variability

Ten patients were randomly selected and remeasured by 2 experienced observers to examine the reproducibility of LA function measurements by RT3DE. Intra-observer variability was assessed by the same observer at different points in time, with an interval of at least 4 weeks between each assessment. Inter-observer variability was performed by the 2 independent observers who repeated the measurements twice.

### Statistical analysis

Statistical analyses were performed using SPSS 21.0 statistical software (SPSS Inc., Chicago, USA). Continuous data were summarized as mean ± standard deviation and categorical variables as numbers and percentages. Normal distribution was analyzed using the Shapiro–Wilk test. Differences in continuous variables between 2 groups were assessed by Student t-test. Comparisons among ≥3 groups were assessed using one-way analysis of variance, and post hoc analysis of variance using Scheffe’s method was performed to compare the differences between groups. Categorical variables were compared by chi-square test. Selections of independent variables for the prediction of CSFP were performed using multivariate analysis. Receiver-operating characteristic (ROC) analysis was used to evaluate the diagnostic effects of the independent variables and to determine the optimal cut-off value. Intra- and inter-observer variabilities were calculated by intraclass correlation coefficient (ICC). *P* < 0.05 was considered statistically significant.

## Results

### Patient clinical characteristics, angiographic findings, and LV function

There were no differences in clinical characteristics between CSFP patients and control subjects **(**Table 1**)**. The TFCs of cLAD, LCX, and RCA, and the mean TFC were significantly greater in the CSFP group than in the control group. Of the 60 CSFP patients, 52 (87%) presented involvement of LAD, 42 (70%) of LCX, and 36 (60%) of RCA. Also, there was one vessel involvement in 23% of the patients, two vessel involvement in 37%, and three-vessel involvement in 40%.

**Table 1:** Clinical characteristics and LV function measurements

	Controls (*n* = 45)	CSFP (*n* = 60)	*P*-value
Clinical characteristics			
Age (yrs)	55.53 ± 8.44	56.52 ± 8.82	0.57
Male sex [n(%)]	21 (47%)	36 (60%)	0.18
Systolic blood pressure (mm Hg)	128.12 ± 14.44	125.94 ± 11.42	0.41
Diastolic blood pressure (mm Hg)	76.71 ± 9.93	75.78 ± 9.91	0.63
Hypertension [n(%)]	20 (44%)	26 (43%)	0.91
Diabetes mellitus [n(%)]	2 (4%)	5 (8%)	0.70
Fasting blood glucose (mmol/L)	5.41 ± 0.87	5.60 ± 0.97	0.29
Triglycerides (mmol/L)	1.41 ± 0.83	1.43 ± 0.70	0.86
Total cholesterol (mmol/L)	4.43 ± 0.80	4.35 ± 0.85	0.90
LDL cholesterol (mmol/L)	2.67 ± 0.82	2.79 ± 0.75	0.45
HDL cholesterol (mmol/L)	1.16 ± 0.30	1.07 ± 0.22	0.10
TFC			
cLAD	21.77 ± 3.23	42.16 ± 16.11	**< 0.001**
LCX	21.47 ± 3.85	35.77 ± 16.53	**< 0.001**
RCA	21.20 ± 4.11	36.82 ± 18.76	**< 0.001**
Mean	21.48 ± 2.27	38.25 ± 13.57	**< 0.001**
LV systolic function measurements			
LV end-diastolic volume (ml)	82.27 ± 15.08	85.05 ± 16.92	0.38
LV ejection fraction (%)	64.27 ± 4.44	63.03 ± 3.96	0.14
LV GLS (%)	−20.58 ± 2.36	−19.24 ± 2.27	**0.004**
LV diastolic function measurements			
LA Vol_max_ index	27.76 ± 7.51	31.83 ± 8.47	**0.03**
Mitral E (m/s)	74.89 ± 15.47	66.67 ± 17.24	**0.01**
Mitral E/A	1.16 ± 0.37	0.96 ± 0.29	**0.003**
Mitral e’ (cm/s)	8.67 ± 2.19	7.72 ± 1.69	**0.01**
Mitral E/e’	8.99 ± 2.26	8.86 ± 2.45	0.78
Tricuspid regurgitation velocity (m/s)	2.01 ± 0.78	2.23 ± 0.89	0.56

Values shown are means±SD or percentages

Abbreviation: *LDL* low-density lipoprotein, *HDL* high-density lipoprotein, *TFC* thrombolysis in myocardial infarction frame count, *cLAD* corrected left anterior descending coronary artery, *LCX* left circumflex coronary artery, *RCA* right coronary artery, *LV* left ventricle, *GLS* global longitudinal strain, *LA Vol*_*max*_ maximal LA volume, *E* early diastolic flow velocity, *A* late diastolic flow velocity, *e’* early diastolic annular velocity

Moreover, we found that CSFP patients had significantly decreased LV GLS compared with control subjects, although there were no significant differences in LVEF or LV end-diastolic volume between groups. Furthermore, CSFP patients presented significantly impaired LV diastolic function when comparing LA volume_max_ index, mitral E, mitral E/A, and mitral e’ between CSFP patients and control subjects.

### LA volume and function

**Fig. 1:**
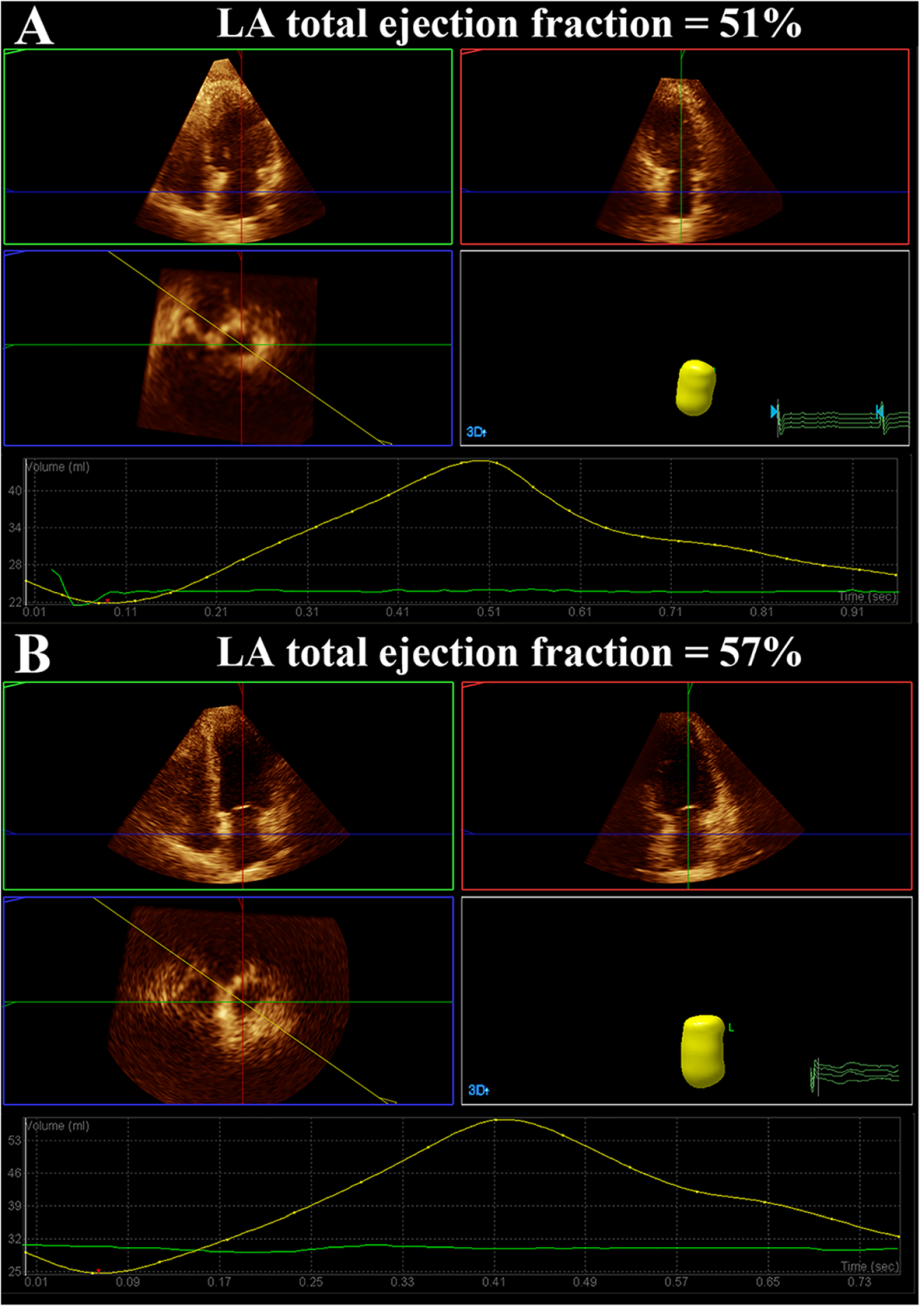
LA total ejection fraction by RT3DE in patients with CSFP **a** and control subjects **b**. Note the reduction of LA total ejection fraction in patients with CSFP compared to that in control subjects. LA, left atrium

The analyses of LA volume showed that CSFP patients had significantly larger LA volume in both 2DE and RT3DE than control subjects, including LA Vol_max_, Vol_p_ and Vol_min_**(**Table 2 and Fig. 1**)**. Also, the analyses of LA function showed that CSFP patients had significantly impaired LA total and active ejection fraction in both 2DE and RT3DE compared to control subjects. However, there was no significant difference in LA passive ejection fraction between groups. Also, there were no significant differences between LA volume and function parameters measured by 2DE and RT3DE in patients with CSFP **(**Table 3**)**. Subsequently, we analyzed the relationships between LA volume and function parameters with LV GLS, mitral E, E/A, and e’ **(**Table 4**)**. The results indicated that LA volume and function were significantly correlated with LV systolic and diastolic function. Furthermore, we evaluated LA phasic volume and function by RT3DE between CSFP patients with normal LVFP and patients with increased LVFP (Table 5). It was showed that there were 50 (83.3%) CSFP patients with normal LVFP, 8 (13.3%) CSFP patients with indeterminate LVFP, 2 (3.3%) CSFP patients with increased LVFP. And LA phasic volume progressively increased among CSFP patients with normal, indeterminate and increased LVFP, but there were no significant differences in LA phasic function among these groups.

**Table 2:** Comparison of LA function measurements

	Controls (*n* = 45)	CSFP (*n* = 60)	*P*-value
LA 2D volumes measurements			
LA 2D Vol_max_ (mL)	47.31 ± 12.31	54.53 ± 13.59	**0.006**
LA 2D Vol_p_ (mL)	32.96 ± 9.69	38.97 ± 11.52	**0.006**
LA 2D Vol_min_ (mL)	20.56 ± 6.25	26.11 ± 9.61	**0.001**
LA 2D total ejection fraction (%)	56.83 ± 6.08	52.52 ± 9.99	**0.001**
LA 2D active ejection fraction (%)	38.74 ± 6.79	33.60 ± 9.77	**0.003**
LA 2D passive ejection fraction (%)	27.64 ± 6.75	26.40 ± 9.57	0.46
LA 3D volumes measurements			
LA 3D Vol_max_ (mL)	49.29 ± 8.23	54.78 ± 10.78	**0.007**
LA 3D Vol_p_ (mL)	34.52 ± 6.11	39.31 ± 8.64	**0.003**
LA 3D Vol_min_ (mL)	21.75 ± 4.89	26.90 ± 7.05	**< 0.001**
LA 3D total ejection fraction (%)	56.40 ± 3.03	51.38 ± 3.85	**< 0.001**
LA 3D active ejection fraction (%)	37.13 ± 6.42	31.78 ± 6.24	**< 0.001**
LA 3D passive ejection fraction (%)	29.94 ± 5.09	28.37 ± 4.57	0.12

Values shown are means±SD

Abbreviation: *LA* left atrium, *2D* two-dimensional, *3D* three-dimensional, *LA Vol*_*max*_ maximal LA volume in ventricular systole just before mitral valve opening, *LA Vol*_*min*_ minimal LA volume after mitral valve closure, *LA Vol*_*p*_ LA volume at the onset of the P wave on electrocardiography

**Table 3:** Comparison of LA two-dimensional and three-dimensional measurements in patients with CSFP

	2D measurements (*n* = 60)	3D measurements (*n* = 60)	*P*-value
LA Vol_max_ (mL)	54.53 ± 13.59	54.78 ± 10.78	0.78
LA Vol_p_ (mL)	38.97 ± 11.52	39.31 ± 8.64	0.38
LA Vol_min_ (mL)	26.11 ± 9.61	26.90 ± 7.05	0.10
LA total ejection fraction (%)	53.34 ± 9.44	51.32 ± 3.86	0.09
LA active ejection fraction (%)	32.90 ± 9.47	31.78 ± 6.24	0.48
LA passive ejection fraction (%)	27.14 ± 9.71	28.37 ± 4.57	0.42

Values shown are means±SD or percentages

Abbreviation: *2D* two-dimensional, *3D* three-dimensional, *LA* left atrium, *LA Vol*_*max*_ maximal LA volume in ventricular systole just before mitral valve opening, *LA Vol*_*min*_ minimal LA volume after mitral valve closure, *LA Vol*_*p*_ LA volume at the onset of the P wave on electrocardiography

**Table 4:** Correlation of LA volume and function by RT3DE with LV systolic and diastolic function

	LV GLS		mitral E		mitral E/A		mitral e’	
	*r*	*P*	*r*	*P*	*r*	*P*	*r*	*P*
LA 2D Vol_max_ (mL)	0.16	0.11	−0.14	0.17	−0.24	**0.01**	−0.32	**0.001**
LA 2D Vol_p_ (mL)	0.18	0.07	−0.24	**0.01**	−0.35	**0.001**	−0.37	**< 0.001**
LA 2D Vol_min_ (mL)	0.22	**0.03**	−0.20	**0.04**	−0.26	**0.009**	−0.31	**0.001**
LA 2D total ejection fraction (%)	−0.18	0.07	0.21	**0.04**	0.20	**0.04**	0.24	**0.01**
LA 2D active ejection fraction (%)	−0.23	**0.02**	0.11	0.25	−0.05	0.60	0.06	0.52
LA 2D passive ejection fraction (%)	−0.15	0.13	0.26	**0.008**	0.27	**0.005**	0.21	**0.03**
LA 3D Vol_max_ (mL)	0.23	**0.02**	−0.06	0.58	− 0.16	0.12	− 0.24	**0.02**
LA 3D Vol_p_ (mL)	0.25	**0.02**	−0.10	0.32	−0.21	**0.04**	−0.29	**0.004**
LA 3D Vol_min_ (mL)	0.29	**0.005**	−0.12	0.25	−0.18	0.09	−0.25	**0.02**
LA 3D total ejection fraction (%)	−0.29	**0.004**	0.21	**0.04**	0.17	0.10	0.20	0.05
LA 3D active ejection fraction (%)	−0.24	**0.02**	0.13	0.21	0.04	0.68	0.06	0.57
LA 3D passive ejection fraction (%)	−0.11	0.30	0.19	0.07	0.21	**0.04**	0.22	**0.03**

Abbreviation: *LA* left atrium, *2D* two-dimensional, *3D* three-dimensional, *LA Vol*_*max*_ maximal LA volume in ventricular systole just before mitral valve opening, *LA Vol*_*min*_ minimal LA volume after mitral valve closure, *LA Vol*_*p*_ LA volume at the onset of the P wave on electrocardiography

**Table 5:** Comparison of LA function by RT3DE between CSFP patients with normal and increased LVFP

	Normal LVFP (*n* = 50)	Indeterminate LVFP (*n* = 8)	Increased LVFP (*n* = 2)	*P*-value
LA 3D Vol_max_ (mL)	51.76 ± 8.12	65.54 ± 8.03	82.00 ± 9.33^*#^	**< 0.001**
LA 3D Vol_p_ (mL)	36.77 ± 6.17	48.67 ± 6.12	61.10 ± 12.02^*#^	**< 0.001**
LA 3D Vol_min_ (mL)	25.05 ± 5.06	33.51 ± 7.45	43.60 ± 10.61^*#^	**< 0.001**
LA 3D total ejection fraction (%)	51.74 ± 3.56	50.40 ± 4.60	47.20 ± 6.93	0.21
LA 3D active ejection fraction (%)	31.96 ± 5.64	31.42 ± 10.16	28.98 ± 3.39	0.80
LA 3D passive ejection fraction (%)	28.94 ± 4.34	25.63 ± 5.15	25.84 ± 6.22	0.15

Abbreviation: *LA* left atrium, *3D* three-dimensional, *LA Vol*_*max*_ maximal LA volume in ventricular systole just before mitral valve opening, *LA Vol*_*min*_ minimal LA volume after mitral valve closure, *LA Vol*_*p*_ LA volume at the onset of the P wave on electrocardiography

^*^P < 0.05 vs. Normal LVFP; ^#^*P* < 0.05 vs. Indeterminate LVFP

### Correlation of LA volume and function parameters with mean TFC and number of involved arteries

**Fig. 2:**
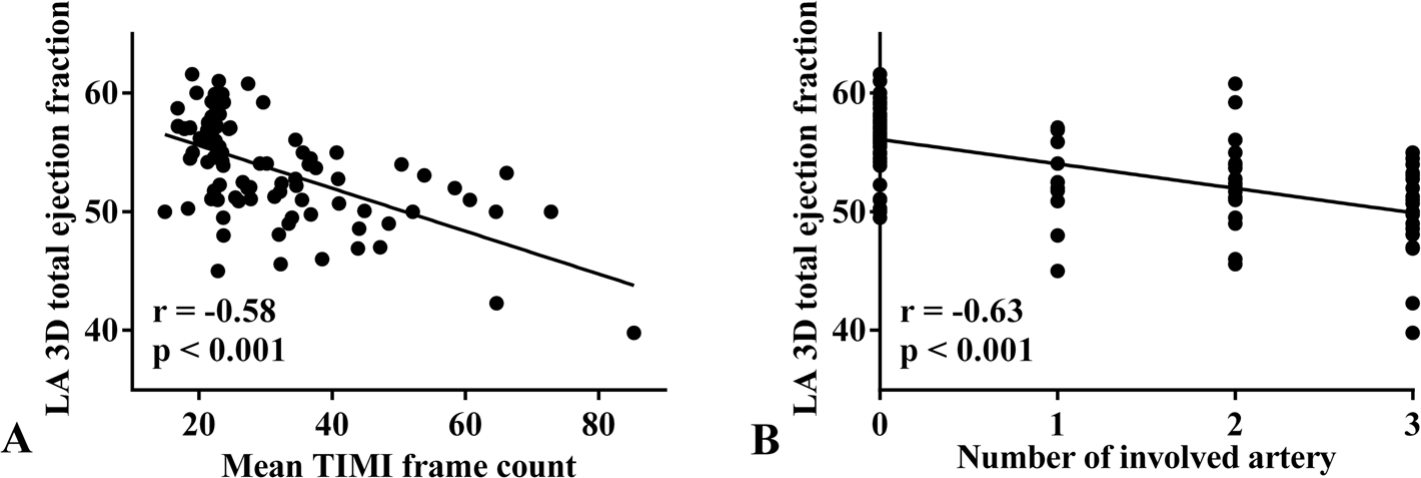
Correlation between LA total ejection fraction by RT3DE with mean TFC and number of involved arteries. LA, left atrium. TFC, TIMI frame count. TIMI, thrombolysis in myocardial infarction

The relationships of LA volume and function parameters with mean TFC and number of involved arteries are presented in Table 6. We found that LA volume parameters, including LA Vol_max_, Vol_p_ and Vol_min_, were positively correlated with mean TFC and the number of involved arteries. However, LA total and active ejection fraction was negatively correlated with mean TFC and number of involved arteries. Also, the highest correlation coefficients were between LA total ejection fraction measured by RT3DE with mean TFC and number of involved arteries **(**Fig. 2**)**.

**Table 6:** Correlation of LA volume and function parameters with mean TIMI frame count and number of involved artery

	Mean TIMI frame count		Number of involved artery	
	r	*P*	r	*P*
LA 2D Vol_max_ (mL)	0.27	**0.007**	0.22	**0.03**
LA 2D Vol_p_ (mL)	0.25	**0.01**	0.19	**0.05**
LA 2D Vol_min_ (mL)	0.29	**0.003**	0.25	**0.01**
LA 2D total ejection fraction (%)	−0.15	0.13	−0.22	**0.02**
LA 2D active ejection fraction (%)	−0.24	**0.02**	−0.32	**0.001**
LA 2D passive ejection fraction (%)	−0.02	0.81	−0.06	0.56
LA 3D Vol_max_ (mL)	0.28	**0.007**	0.25	**0.01**
LA 3D Vol_p_ (mL)	0.32	**0.002**	0.27	**0.009**
LA 3D Vol_min_ (mL)	0.41	**< 0.001**	0.42	**< 0.001**
LA 3D total ejection fraction (%)	−0.58	**< 0.001**	−0.63	**< 0.001**
LA 3D active ejection fraction (%)	−0.35	**< 0.001**	−0.43	**< 0.001**
LA 3D passive ejection fraction (%)	−0.16	0.11	−0.17	0.10

Abbreviation: *LA* left atrium, *2D* two-dimensional, *3D* three-dimensional, *LA Vol*_*max*_ maximal LA volume in ventricular systole just before mitral valve opening, *LA Vol*_*min*_ minimal LA volume after mitral valve closure, *LA Vol*_*p*_ LA volume at the onset of the P wave on electrocardiography

### Predictors of CSFP

Logistic regression analysis showed that LA total ejection fraction measured by RT3DE was the only independent predictor for CSFP, after adjusting for age, sex, and other covariates such as LV GLS, LV diastolic function measurements, and LA function measurements, with *P* < 0.05 in the univariate model (odds ratio, 0.64 [95% confidence interval (CI), 0.49–0.83]; *P* = 0.001) **(**Table 7**)**.

**Table 7:** Prediction of coronary slow flow phenomenon

	Univariate analysis		Multivariate analysis	
	OR [95% CI]	*P*-value	OR [95% CI]	*P*-value
Age	1.01 [0.97–1.06]	0.56	0.96 [0.89–1.03]	0.26
Sex	1.71 [0.79–3.74]	0.18	1.13 [0.31–4.09]	0.85
LV global longitudinal strain (%)	1.28 [1.08–1.53]	**0.006**	1.21 [0.93–1.57]	0.15
Mitral E (m/s)	0.97 [0.95–0.99]	**0.02**	1.03 [0.98–1.08]	0.29
Mitral E/A	0.15 [0.04–0.53]	**0.003**	0.06 [0.003–1.05]	0.05
Mitral e’ (cm/s)	0.77 [0.61–0.96]	**0.02**	0.93 [0.63–1.37]	0.70
LA 2D total ejection fraction (%)	0.94 [0.89–0.99]	**0.02**	1.06 [0.97–1.15]	0.24
LA 2D active ejection fraction (%)	0.93 [0.89–0.98]	**0.005**	0.93 [0.86–1.001]	0.05
LA 3D total ejection fraction (%)	0.65 [0.54–0.77]	**< 0.001**	0.64 [0.49–0.83]	**0.001**
LA 3D active ejection fraction (%)	0.87 [0.80–0.94]	**< 0.001**	1.02 [0.90–1.15]	0.78

Abbreviation: *LV* left ventricle, *E* early diastolic flow velocity, *A* late diastolic flow velocity, *e’* early diastolic annular velocity, *LA* left atrium, *2D* two-dimensional, *3D* three-dimensional

**Fig. 3:**
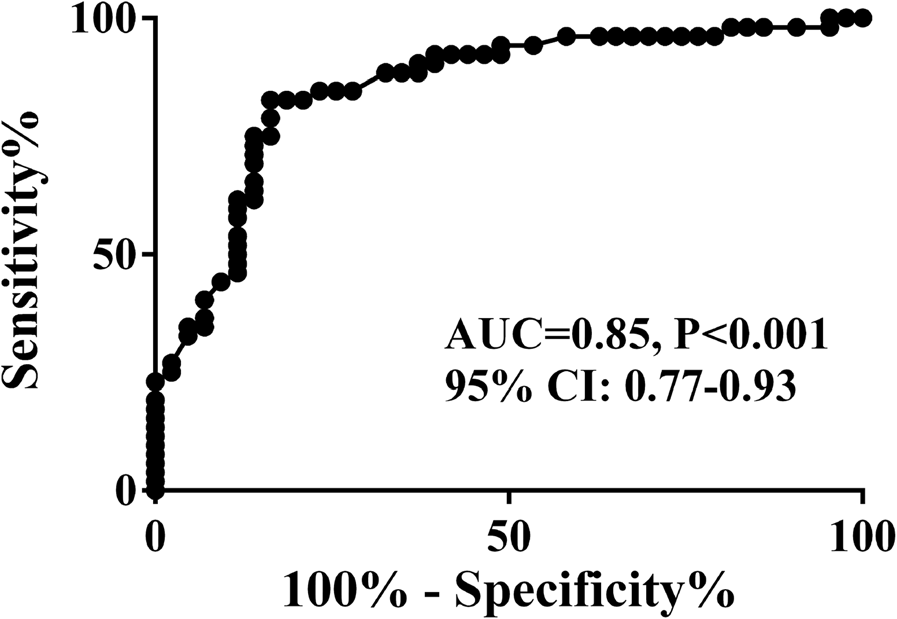
Receiver-operating characteristic curve analysis of LA total ejection fraction by RT3DE as predictive tool for CSFP. LA, left atrium

ROC (receiver operating curve) analysis indicated that LA total ejection fraction measured by RT3DE presented good prediction power for CSFP (area under curve, 0.85; sensitivity, 84%; specificity, 83%) and cut-off value was 54.15% **(**Fig. 3**)**.

### Intra- and inter-observer variability

LA total ejection fraction, active ejection fraction, and passive ejection fraction measured by RT3DE showed excellent intra-observer correlation with ICC values 0.95 (95% CI, 0.90–0.98; *P* < 0.001), 0.94 (95% CI, 0.89–0.98; *P* < 0.001) and 0.94 (95% CI, 0.89–0.98; *P* < 0.001), and excellent inter-observer correlation with ICC values 0.94 (95% CI, 0.89–0.98; *P* < 0.001), 0.92 (95% CI, 0.85–0.97; *P* < 0.001) and 0.91 (95% CI, 0.84–0.97; *P* < 0.001).

## Discussion

CSFP is characterized by the delayed opacification of the coronary distal vessel in the absence of any obstructive lesions in the epicardial coronary artery. It has been revealed that CSFP patients may experience recurrent angina or repeat coronary angiograph, which severely affects the quality of life of the patients [15]. Therefore, it is extremely important to determine the severity, development or even prognosis of CSFP. The LA function has been recognized as a powerful marker to estimate prognosis or for further prevention of a diversity of cardiovascular diseases [8]. Therefore, we evaluated the LA function by RT3DE in patients of CSFP in the present study. Our results indicated LA total and active ejection fraction was decreased and correlated with coronary flow rate and the number of involved arteries, and LA total ejection fraction by RT3DE was the only independent predictor for CSFP.

Echocardiography is the most common non-invasive imaging technique used to quantify LA volume and function. Although conventional 2DE frequently uses the biplane method, its measured value may be affected due to the significant geometric assumptions about LA nonsymmetrical shape or foreshortening of LA cavity in the apical views [16]. In comparison, RT3DE can more accurately assess LA volume, without the limitations of conventional 2DE [17]. Moreover, it has been revealed that LA volume assessment by RT3DE has favorable test-retest variation, which was consistent with the analysis results of intra- and inter-observer variability in our study. In addition to LA volume, recent studies used tissue Doppler imaging and two-dimensional strain to assess LA function [18, 19]. However, it should be noted that tissue Doppler imaging has angle dependence, and the two-dimensional strain value may be influenced by the lack of dedicated software algorithm for LA strain, inability of capturing a region of interest resembling LA nonsymmetrical shape, varying interpolation across pulmonary vein orifices, and the LA appendage, uncertain atrial septal hypertrophy, or confounding effects on the echo signal by structures surrounding the left atrium [20]. In contrast, RT3DE provides a unique combination of accurate measurement of LA volumes, with rapid and automatic detection of LA phasic function [21, 22]. For all these reasons, RT3DE was chosen to evaluate LA volume and function in the present study.

In our study, in order to demonstrate the independent effect of CSFP on LA function in a limited population, we designed the case control study and enrolled both the CSFP patients and the controls without CSFP matched by age, sex, blood pressure and other clinical characteristics to control the confounding factors, and we found that LA total and active ejection fraction decreased in patients with CSFP, which indicated that LA reservoir and contractile function was impaired. The mechanisms underlying the effect of CSFP on LA function might be explained by the following inferences. First, CSFP may lead to chronic LA myocardial hypoperfusion, which may further impair LA contractility or decrease LA compliance due to secondary LA fibrosis or LA myopathy. Second, the LA function has phase roles throughout cardiac cycle and plays strong interactions with LV in each phase of LA function. The impaired LA function may be related to the decreased LV systolic and diastolic function, which is evidenced by our results. Due to the interactions between decreased LA and LV function, a vicious loop may be generated and, ultimately, cause decompensation of atrial function and decreased cardiac output [23]. Therefore, monitoring changes in atrial function in patients with CSFP may provide a sensitive method for a timely clinical intervention. Moreover, in addition to improvement of LV function by improving myocardial perfusion, unloading the LA, and improving its function can also break the vicious loop, and may be another important therapeutic endpoint which should be validated by another study in the future [24].

Additionally, we also found that LA phasic function correlated with coronary flow rate and the number of involved arteries, and LA total ejection fraction by RT3DE was the only independent predictor for CSFP. These results suggest that LA dynamic abnormalities may be observed at an earlier stage than LV diastolic dysfunction, and further emphasized the incremental value of RT3DE for evaluating LA function in patients with CSFP compared to 2DE. Moreover, these findings also suggest that slower coronary flow and a greater number of involved vessels represent more severe lesions in patients with CSFP. These patients should be closely monitored in long-term follow-up, and LA total ejection fraction by RT3DE could constitute a simple and noninvasive alternative for determining prognosis in the follow-up period. However, the level of feasibility and reliability warrants further prospective studies with larger sample sizes.

Recently, Xing et al. [25] reported that CSFP patients had impaired LA reservoir function and enhanced contractile function using RT3DE, but the incremental value of RT3DE for assessing LA function in patients with CSFP is not clear. Notably, our study showed that both LA reservoir and contractile function was impaired, and further researched the incremental value of RT3DE compared to 2DE for evaluating LA function in patients with CSFP, and confirmed the diagnostic effects for CSFP of LA total ejection fraction by RT3DE and its cut-off value (54.15%), which may provide a reliable reference basis for the evaluation of prognosis, disease severity or even therapeutic efficacy in the patients with CSFP.

## Limitations

The major limitation of our study was attributed to the low frame rates achieved by the X3–1 transducer. In our study, we attempted to increase the frame rate by narrow width and minimum depth and avoided using frame rates lower than one third of the heart rate. Moreover, the exclusion of patients with arrhythmia due to technical limitations of RT3DE limits the generalizability of our findings in CSFP patients with arrhythmia.

Another study limitation was the lack of invasive hemodynamic and histological data, which may provide more accurate information about LA pressure and provide clear basis for the contribution of LA fibrosis or other LA myopathy secondary to CSFP in LA dysfunction.

In addition, the sample size of the study population may not be adequate, which may be due to the high selection of the population to control confounding factors in LA evaluation and the low prevalence of CSFP. Thus the results was based on a hyper-selected population with a rather low average age. Therefore, we are expanding the sample size and performing a follow-up of these patients to validate these findings.

## Conclusions

LA reservoir and contractile function were decreased in the patients with CSFP and correlated with coronary flow rate and the number of involved arteries. LA total ejection fraction by RT3DE can independently predict CSFP, and RT3DE demonstrated incremental value for evaluating LA phasic function compared to 2DE. These findings may provide a theoretical basis for the evaluation of prognosis, disease severity or even therapeutic efficacy in patients with CSFP.

## Data Availability

The datasets during and/or analyzed during the current study is available from the corresponding author on reasonable request.

## Abbreviations

2DE: Two-dimensional echocardiography
ASE: American Society of Echocardiography
CSFP: Coronary slow flow phenomenon;
cTFC: Corrected TIMI frame count
GLS: Global longitudinal strain
ICC: Intraclass correlation coefficient
LA: Left atrial
LAD: Left anterior descending coronary artery
LCX: Left circumflex coronary artery
LV: Left ventricular
LVEF: LV ejection fraction
RCA: Right coronary artery
ROC: Receiver-operating characteristic
RT3DE: Real-time three-dimensional echocardiography
TFC: TIMI frame count
TIMI: Thrombolysis in myocardial infarction
Vol_max_: Maximal volume
Vol_min_: Minimal volume
Vol_p_: Pre-systolic volume
